# Knock-Down of Glucocorticoid Receptor Beta (GRβ) Enhances the Ocular Hypertensive Response to Dexamethasone in C57BL/6J Mice

**DOI:** 10.3390/ijms27198483

**Published:** 2026-09-23

**Authors:** J. Cameron Millar, Pinkal D. Patel, Abbot F. Clark

**Affiliations:** Department of Pharmacology and Neuroscience, The North Texas Eye Research Institute, University of North Texas Health Fort Worth, 3500 Camp Bowie Boulevard, Fort Worth, TX 76107, USA; pinkal.patel@unthsc.edu (P.D.P.); abe.clark@unthsc.edu (A.F.C.)

**Keywords:** ocular hypertension, glaucoma, mouse, mouse models, dexamethasone, GRβ, glucocorticoids, IOP

## Abstract

Topical ocular treatment with glucocorticoids (GCs) yields an increase in intraocular pressure (IOP) in a subset (~35–40%) of the human population, known as steroid responders. If treatment is continued, this ocular hypertension (OHT) can develop into GC-induced glaucoma. In mice, the GC-mediated OHT response is modulated by the GC receptor, which exists as two major splice variants, GRα and GRβ, in the TM cell cytosol. GRα contains a GC-binding domain. Following GC binding, the GRα-GC complex enters the TM cell nucleus and transactivates genes associated with increased synthesis/export of extracellular matrix (ECM), causing OHT. GRβ has no ligand-binding domain but appears to inhibit gene transactivation by the GRα-GC complex. We previously described the effects of over-expression of GRβ on the GC-induced OHT response on IOP in mice. Here, we investigated the effect of the GC Dexamethasone (DEX) on the OHT response in C57BL/6J mice, under conditions of GRβ knockdown. A total of 20 × C57BL/6J mice (♀), aged 3–4½ months were used. Animals were weighed. Baseline (BL) conscious IOP measurements (TonoLab) and direct ophthalmoscopy (Keeler Instruments Specialist Model Ophthalmoscope) assessments were made. Animals were divided into 4 cohorts (n = 5/cohort), and anesthetized (isoflurane (2.5%) and O_2(g)_ (0.8 L/min)). Cohorts 1 and 2 (n = 10 animals) were given an intravitreal (IVT) injection in both eyes (OU) of Lenti.shRNA.Scramble (2.2 × 10^9^ TU/mL; 1 μL bolus injection (total 2.2 × 10^6^ TU/eye)). Cohorts 3 and 4 (n = 10 animals) were given an IVT injection OU of Lenti.shRNA.GRβ (1.09 × 10^9^ TU/mL; 2 μL bolus injection (total 2.18 × 10^6^ TU/eye)). Each subsequent week, for 8 weeks, conscious IOPs were measured (OU), and direct ophthalmoscopy was performed. Animals were then anesthetized and given a periocular injection OU of a 20 μL bolus (per eye) of either vehicle solution for DEX (DEX-Veh) (Cohorts 1 (n = 5 animals) and 3 (n = 5 animals), or micronized DEX suspension (Cohorts 2 (n = 5 animals) and 4 (n = 5 animals)). At the conclusion of the study, animals were euthanized and TM tissues were collected (OD and OS pooled per animal) for assay of GRβ mRNA expression using QPCR. GRβ mRNA expression in the TM was significantly reduced by greater than 95% in the Lenti.shRNA.GRβ group vs. the Lenti.shRNA.Scramble group (*p* < 0.0001). Injection of Lenti.shRNA.Scramble or Lenti.shRNA.GRβ had no effect on baseline IOP. Subsequent injections of DEX over the following 8 weeks led to a significant increase in mean IOP in both groups (baseline 12.5 mmHg; 23 mmHg in Lenti.shRNA.Scramble cohort (*p* < 0.0001); and 28 mmHg in Lenti.shRNA.GRβ cohort (*p* < 0.0001)). DEX-induced IOP was significantly higher in the shRNA.GRβ cohort compared to the shRNA.Scramble cohort (*p* < 0.0001). Animals given vehicle control did not show an increase in IOP. Eyes that received DEX had very mild lenticular cataracts at Week 8, but there was no significant reduction in body weight. GRβ plays a role in modulating the IOP response to DEX in C57BL/6J mice. Knockdown of endogenous mRNA encoding GRβ results in an enhanced IOP response to DEX.

## 1. Introduction

Glaucoma is a heterogenous group of optic neuropathies that share the clinical phenotype of progressive axonopathy of the optic nerve, cupping of the optic nerve head, and death of retinal ganglion cells. Glaucoma continues to be a leading cause of irreversible vision loss and blindness in the world, and it is estimated that approximately 190 million individuals will develop glaucoma by 2026 [1]. There are numerous rodent models to study the various aspects of glaucoma, and each model has its own strengths and limitations [2,3,4,5,6]. Many models directly damage the optic nerve (ON) (e.g., ON crush or transection) [7,8,9] or the inner retina (e.g., ischemia/reperfusion injury or exitotoxicity injury) [7,10,11] to test potential neuroprotective agents. Other models artificially block/damage the aqueous outflow pathway, thereby elevating intraocular pressure (IOP), which causes glaucomatous optic neuropathy [9,12,13]. Understanding glaucomatous pathogenic signaling in the human trabecular meshwork (TM) has led to more relevant rodent models of glaucomatous ocular hypertension (OHT) (e.g., TGFβ2- or GREM1-induced OHT) [14,15,16]. We have chosen the highly relevant mouse model of glucocorticoid-induced OHT (GC-OHT) that very closely mimics all the features of GC-OHT and glucocorticoid-induced glaucoma (GIG) in man [17,18,19,20,21] to discover the molecular mechanisms responsible for GC damage to the TM and aqueous humor outflow pathway.

Since the 1940s, synthetic topical ocular glucocorticoids (GCs) lacking mineralocorticoid activity have become one of the most widely prescribed classes of anti-inflammatory and immunosuppressive drugs in the world. It is estimated that each year between 1 and 3% of the world’s population receives GC therapy [22,23,24,25]. GCs are used as a primary source of therapy for suppression of inflammation in a variety of diseases, including (but not limited to): hyper-reactive airway disease, Grave’s disease, and autoimmune diseases. GCs are also used to treat inflammatory eye diseases, for example: conjunctivitis, keratitis, optic neuritis, and uveitis. However, chronic GC use leads to numerous side-effects, contributing to both health issues and a collective economic burden [26]. There are several significant ocular side-effects associated with the chronic use of GCs, including posterior subcapsular cataract, as well as elevation of IOP that can cause the development of iatrogenic glaucoma [27,28,29].

One of the problems associated with the use of GCs in the eye as anti-inflammatory therapy is approximately 35–40% of individuals receiving ocular GC therapy develop increased IOP. These individuals are known as steroid responders. The remaining 60 to 65% individuals who do not develop OHT are classified as steroid non-responders [25,26,30,31,32,33]. In steroid responders, treatment with GCs initially causes GC-OHT, and prolonged treatment can lead to GIG. In GC responders, the chronic use of GCs in the eye leads to this iatrogenic form of secondary open-angle glaucoma by promoting extracellular matrix (ECM) deposition within the TM [34,35,36] which increases aqueous humor outflow resistance, thereby elevating IOP. The TM is a small, reticulated tissue at the iridocorneal angle that regulates aqueous humor outflow by imparting a homeostatic resistance to this outflow to maintain the spherical shape of the eyes. Similar in many ways to primary open-angle glaucoma (POAG) [28], prolonged OHT in GIG leads to progressive death of retinal ganglion cells (RGCs) and their axons, leading to progressive loss of vision. It is also of note that GC-induced OHT is considerably more commonplace in individuals who already have POAG [30,37,38].

We previously reported a reliable and reproducible model of GC-OHT in C57BL/6J mice [18,19,20]. In this model, weekly bilateral periocular injections of the potent GC dexamethasone (DEX) are administered, and after 1–4 weeks IOP elevation occurs. This OHT persists as long as weekly injections are maintained, for at least 20 weeks [21]. In the C57BL/6J mouse, the GC-mediated ocular hypertensive (OHT) response is modulated by GC receptors in the TM cell cytosol as two major alternatively spliced variants, GRα and GRβ [39,40,41]. GRα contains the GC ligand-binding domain and mediates the numerous biological and pharmacological effects of GCs. The alternatively spliced isoform GRβ lacks the GC-binding domain in exon 9 and acts as a dominant negative regulator of GC activities [26,42,43]. Following GC binding to GRα, the GRα–GC complex translocates to the cell nucleus, where this homodimer directly activates numerous genes (in a process known as transactivation) including genes associated with increased synthesis and deposition of extracellular matrix (ECM) proteins within the TM [34,35,36], thus causing OHT [19]. Activated GRα can also bind to and inhibit the activities of other transcription factors (e.g., AP-1, NFκB) in a process called transrepression [17,43].

GRβ inhibits gene activation by the GRα-GC homodimer complex. We previously reported that GRβ acts as a dominant negative regulator of GR activity in the TM in vitro [39,41,44,45,46] and in vivo [18]. We showed that over-expression of GRβ in the TM of C57BL/6J mouse eyes treated with DEX totally reversed the DEX-induced IOP elevation and increased aqueous humor total outflow facility, which corresponded with the measured reduction in IOP. GRβ also inhibited DEX effects on TM cells in vitro by preventing DEX-induced expression of fibronectin, collagen 1, and myocilin [20,44,46] as well as reversing DEX-mediated inhibition of phagocytosis [44]. However, we previously have not studied the effects of the reduction of endogenous GRβ in DEX-treated animals. Thus, in the present study, we examined the effects of knock-down of GRβ using Lenti.shRNA.GRβ on the DEX GC-OHT response in C57BL/6J mice.

## 2. Results

To confirm shRNA mediated knock-down of GRβ in the TM of mouse eyes, we quantified GRβ mRNA expression. QPCR demonstrated a statistically significant >95% reduction in GRβ mRNA expression (GRβ expression normalized to 18S RNA; *p* < 0.0001) (Figure 1). We validated our GRβ mRNA knockdown results in the same samples using an independent set of GRβ PCR primers. We have previously used Lentivirus delivery to the eye and demonstrated strong tropism for the TM vs. other ocular tissues.

Lenti.shRNA.Scramble- or Lenti.shRNA.GRβ vehicle (Veh)-treated groups had no effect on conscious IOPs (Figure 2). Subsequent DEX injections once per week for 8 weeks led to a mean increase in conscious IOP of 10.5 mmHg in the Lenti.shRNA.Scramble cohort and a greater mean increase in conscious IOP of 15.5 mmHg in the Lenti.shRNA.GRβ cohort (**** *p* < 0.0001 for both DEX groups compared to vehicle Veh controls). Knockdown of GRβ in the DEX + shRNA.GRβ group caused an additional significant increase in IOP compared to the DEX + shRNA.Scramble control group (Figure 2; #### *p* < 0.0001).

We measured in vivo total aqueous humor outflow facilities (C_tot_) in all four groups after 8 weeks of treatment (Figure 3). There was no significant difference in C_tot_ values between both vehicle groups treated with the Lenti.shRNA vectors (22 vs. 24 nL/min/mmHg; NS). C_tot_ was significantly reduced in the DEX + shRNA.GRβ group (*p* < 0.005 vs. Veh + shRNA.Scramble; *p* < 0.015 vs. Veh + shRNA.GRβ). C_tot_ also showed an apparent reduction in the DEX + shRNA.Scramble group (17 vs. 24 nL/min/mmHg), but this just missed statistical significance (*p* = 0.057 vs. Veh + shRNA.Scramble).

An additional clinical side-effect of ocular GC treatment in human eyes is the development of posterior subcapsular cataracts. Therefore, we examined our mice for cataracts by direct ophthalmoscopy. The DEX-injected eyes exhibited very mild nuclear or cortical cataracts following 8 weeks of bilateral periocular injections of DEX, but we did not observe any evidence of posterior subcapsular cataracts in any DEX-injected eye. The DEX-vehicle-treated mice did not develop lenticular cataracts (Figure 4). Lenticular cataract severity was scored according to an ordinal scale as defined in Table 1.

Systemic exposure to GCs can cause progressive losses in body weight, especially in rodents [35,47,48]. We therefore wanted to determine whether we had significant systemic DEX exposure in our mice receiving weekly periocular injections, as determined by weight loss. Weekly injections of DEX did not lead to significant loss of body weight ≥ 10% in any group of animals (Figure 5). It is possible that this route of DEX exposure caused more subtle systemic effects (e.g., disruption of the HPA axis, leukopenia, etc.), which we did not assess.

## 3. Discussion

We have shown that knockdown of endogenous GRβ in the mouse TM further exacerbates DEX-OHT in B6 mice (Figure 2 and Figure 6). Lentiviral delivery of shRNA targeting GRβ significantly decreased GRβ mRNA expression in the TM. DEX mediates enhanced deposition of ECM in the TM [34,35,36,49], and it is likely that GRβ knockdown further exacerbated this effect, which may be responsible for the apparent further decrease in AH outflow and the additional significant IOP elevation seen in the current study. This clearly indicates that endogenous GRβ continues to regulate DEX signaling within the TM thereby regulating IOP. It is interesting to note the molecular pathogenic similarities between GC-OHT/GIG and POAG [28,50]. For example, a recent whole-exome sequencing study reported enriched ECM organization in POAG [51].

Elevated IOP in steroid responders following chronic treatment with GCs can lead to the development of GIG. Although this observation was first recorded many years ago [30,31,32,38], the precise mechanism(s) underlying this ocular hypertension in response to GCs has remained poorly understood. Patients receiving chronic GC therapy should be closely monitored for the development of OHT. They can then be subsequently treated with IOP-lowering topical ocular medications, laser procedures (trabeculoplasty), and/or glaucoma surgeries, to avoid the development of iatrogenic glaucoma [29,52].

The GC receptor resides within the TM cell cytosol as two major alternatively spliced variants, known as GRα and GRβ. GRα containing the GC-binding domain binds to several heat shock proteins (HSPs) [53] and immunophilins [54]. Following GC binding, the activated GRα–GC dissociates from the HSPs and immunophilins and is actively transported along microtubules to the nuclear membrane where it enters the cell nucleus through nuclear pores [26,53]. Once inside the nucleus, the activated GRα-GC monomers form homodimers, which then bind to palindromic GC response elements (GREs) promoters within GC-regulated genes, leading to increased gene transcription. This process is known as transactivation. In TM cells, this transactivation leads to increased synthesis and deposition of extracellular matrix (ECM) proteins. Increased ECM deposition within the TM along with other changes to the TM cell increases aqueous humor outflow resistance and promotes OHT [17,28,50] (Figure 7).

Activated nuclear GRα-GC monomers can also bind to several other transcription factors (TFs) including AP1 and NFκB, thus preventing these same TFs from binding and activating their own response elements (TREs), in a process known as transrepression. Indeed, many of the anti-inflammatory properties of GCs are mediated through transrepression [17,55] (Figure 7).

The GRβ splice variant of the GC receptor has no ligand-binding domain and inhibits gene transactivation by the GRα-GC complex [42,56] including in the TM [39] (Figure 7). The GRβ alternatively spliced variant isoform is present in human cells [42,56] including TM cells [39]. It is also present in various tissues in rat [57], mouse [58], and zebrafish [59]. However, the alternative splicing mechanisms of the GR receptor to yield GRβ varies amongst these different species. The alternative splicing takes place in the regions of exons 9α and 9β in humans 9 [42,56]. In these other species, the splice domain is located within the intron separating exons 8 and 9 [57,58,59]. But despite this, the GRβ isoforms produced in these species are functionally homologous, and all act as dominant negative regulators of GC activities.

We previously reported that over-expression of the GRβ isoform in the TM of the C57B/6J mouse treated weekly with periocular injections of DEX inhibited DEX-induced OHT and inhibited the DEX-induced reduction in C_tot_ [18]. In our present study, we found that knock-down of endogenous GRβ further enhances the DEX-mediated OHT. The mechanism likely involved a decrease in C_tot_; however, the C_tot_ data were statistically slightly underpowered (the DEX + shRNA.Scramble group was not significantly different from the Veh groups). It was somewhat of a surprise to find that injection of Lenti.shRNA.GRβ followed by weekly periocular injections of vehicle control had no significant effect on basal IOP. We anticipated that GRβ was also involved in homeostatic control of IOP. Perhaps in these animals their level of expression of natural GCs was too low for knock-down of GRβ to lead to any effect on basal IOP.

There was not a significant weight loss of ≥10% in any mouse throughout the course of this study associated with local DEX delivery to the eye (Figure 5). However, other routes of DEX delivery have shown significant losses in mouse body weights [35,47]. We did see the development of very mild cataracts in the DEX-injected animals, which is a major ocular side-effect of prolonged GC therapy in man [60,61] (Figure 4). These mild DEX-induced cataracts in mouse eyes appeared to be nuclear and/or cortical, which is different from the GC-induced PSC cataracts seen in man. This could be due to human vs. murine species differences in lens anatomy and/or the shorter generation time in mice (8 weeks) vs. man (multiple months-years).

Limitations of the present study include the following: (1) the use of only female animals. However, in our hands, previous studies of the effect of DEX on IOP have not shown any evidence of sex differences. But nevertheless, future studies using male alongside female animals are warranted. (2) Lenticular cataract assessments were subjective and made by un-blinded observers (JCM, PDP). (3) The injected viral vector bolus volumes were 1 μL for the Lenti.shRNA.Scramble vector but 2 μL for the Lenti.shRNA.GRβ vector. We utilized this approach to take account of the difference in titer of each vector preparation such that each injected eye would receive a similar number of viral particles. We acknowledge that this was another limitation of the study, but in our hands, such a small difference in bolus volume for a murine intravitreal injection has not led to any known effect on IOP or C_tot_ measurements in of itself. We do not believe that this small volume difference would have any effect on the observed results.

## 4. Materials and Methods

### 4.1. Animals

A total of 20 × C57BL/6J mice (♀, 3–4½ months of age) were obtained from the Jackson Laboratory (Bar Harbor, ME, USA JAX Ref: 000664). All animal experimental studies were conducted in accordance with the ARVO Statement for the Use of Animals in Ophthalmic and Vision Research, and the University of North Texas Health Fort Worth Institutional Animal Care and Use Committee (UNT Health IACUC-2023-0027, approved 24 July 2023). Animals were housed under standard controlled conditions of temperature (21 to 26 °C), humidity (40% to 70%), and a 12 h light/12 h dark cycle (lights on at 7:00 am.). Food (standard mouse chow) and water were provided ad libitum.

Mice were randomly assigned to 4 individual experimental groups (5 animals per group) from the original cohort of 20 C57BL/6J mice. No allocation concealment was utilized. Investigators were not blinded in these studies. Any mouse that exhibited evidence of ill health (failure to react normally to cage mates or human presence, head-tilting, unkempt fur, fur loss, or signs of skin lesions, failure to eat normal chow or drink water) was excluded from the study. Only animals whose eyes appeared normal (by direct ophthalmoscopy) (Keeler Instruments Specialist Model Ophthalmoscope, Malvern, PA, USA) were included (normal appearance of cornea in terms of transparency and with no sign of congestion, discharge, lesions, epithelial abrasions, absence of focal opacities; normal appearance of iris, pupils equal in diameter (OD v. OS) round and reactive to light with normal light reflex (PERRL), no sign of iritis or synechia(e), normal appearance of visible part of crystalline lens with no visible sign of cataract). During the study itself, animals were weighed once per week. Per IACUC directives, any animal that lost more than 10% of body weight at any point was to be removed from the study and treated by veterinary staff. Any animal that lost more than 20% of body weight was to be removed from study and euthanized. Any animal that exhibited signs of ill health was to be removed from the study and treated by veterinary staff. However, no animals were removed from the study due to weight loss or ill health considerations.

### 4.2. Baseline Conscious IOP Measurements

All baseline conscious IOP measurements were made between 11:30am and 1:00pm to minimize natural circadian IOP fluctuation issues. Animals were weighed to obtain their baseline (BL) body weights. BL conscious IOP measurements (TonoLab impact tonometer, Colonial Medical Supply Company, Inc., Londonderry, NH, USA) and direct ophthalmoscopy assessments were then made, as previously described [16,17,18]. Briefly, for tonometry, conscious mice were restrained in a polyethylene cone (Decapicone, Braintree Scientific, Inc., Braintree, MA, USA) sized for use with mice. The narrow end of the cone had first been trimmed with scissors so that the animal’s face could comfortably protrude, but while its ears remained snugly inside the cone. The cone-restrained mouse was then placed in a commercially obtained mouse restrainer (Colonial Medical Supply Company Inc., Londonderry, NH, USA) (Mouse Version MR01C). The Velcro straps were then positioned over the animal. Care was taken to avoid application of pressure to the animal’s thorax, which could potentially increase central venous pressure and thus artificially increase IOP. The animal was placed on top of an adjustable height platform (Lab Jack, Lakewood, CO, USA), and the tip of a TonoLab tonometer (set on the mouse calibration) was brought within 2–3 mm of the central cornea of the eye in which IOP was to be measured. With the probe addressing the central cornea perpendicularly, 6 complete IOP readings were taken (each reading consisting of 6 individual probe strikes which were then averaged automatically by the instrument). Upon completing the IOP readings in one eye, the animal in its restraint was then rotated 180° and a further set of readings was taken in the contralateral eye. Following IOP measurements, both eyes of each animal were examined quickly by direct ophthalmoscopy. Following IOP measurement, mice were released from restraint and returned to their cages.

### 4.3. Measurement of Total Aqueous Humor Outflow Facilities in Living Mice

Total aqueous humor outflow facilities (C_tot_) were measured in anesthetized living mice using our previously published method of constant-flow infusion [62,63]. Briefly, after obtaining the final IOP measurements, mice were anesthetized with ketamine/xylazine (100/10 mg/kg), placed on a 37 °C heating pad, and continuously checked to maintain the required plane of anesthesia via maintenance doses throughout the procedure. Topical anesthesia (one drop of 0.5% proparacaine HCl (Alcaine)) was administered to each eye prior to cannulation with a 13 mm 32-gauge needle attached to the perfusion system previously described [62,63]. The infusion pump stepwise-increased the flow rates from 40, 80, 120, 160, and 200 nL/min. At each step, 15–30 min were allowed for pressure to stabilize, thus avoiding confounding effects of ocular compliance. C_tot_ values were calculated as the reciprocal of the slope of each pressure-flow curve.

### 4.4. Generation of Lentiviral Vectors

Lentiviral vectors Lenti.shRNA.mGRβ (VB220601-1311htw) and Lenti.shRNA.Scramble (VB010000-0009mxc) were commercially sourced from VectorBuilder Inc. (Chicago, IL, USA). The shRNA for targeting mouse GRβ was designed using previously reported oligonucleotide sequence GGACTCCATGCATGATGTAAGTACCAAACATCAAGAGTGTTTGGTACTTACATC-ATGCATGGAGTCTTTTTT [64]. The shRNA targeting sequence was determined to recognize part of exon 8 in the mouse GRβ mRNA (Accession number HM236293.1) and extended into intron 8 (Supplementary Figure S1). The complete shRNA sequence consisted of the targeting sequence, loop sequence, and reverse-compliment targeting sequence was incorporated into the lentiviral plasmid (Supplementary Figure S1) expressed under the control of the U6 promoter. Lentiviral vector expressing scramble shRNA was used as control (Supplementary Figure S2).

### 4.5. Injection of Lenti.shRNA.GRβ or Lenti.shRNA.Scramble

Animals were divided into 4 cohorts and anesthetized with inhalation anesthesia (isoflurane 2.5%/O_2(g)_ 0.8 L/min). Cohorts 1 and 2 (n = 5 animals each) were given intravitreal (IVT) injections to both eyes (OU) of Lenti.shRNA.Scramble (2.2 × 10^9^ TU/mL; 1 μL bolus; total 2.2 × 10^6^ TU/eye). Cohorts 3 and 4 (n = 5 animals each) were given IVT injections OU of Lenti.shRNA.GRβ (1.09 × 10^9^ TU/mL; 2 μL bolus; total 2.18 × 10^6^ TU/eye). Injections were delivered from a glass microsyringe (Hamilton Company) fitted with a 33G steel needle. The eye was proptosed, and then the tip of the needle was passed through the scleral equator such that it entered the vitreous chamber without striking the posterior lens capsule or the retina. The bolus was then injected over the course of 15–30 s. Following injection, the needle was left in place for a further 30 s, before the needle was then rapidly withdrawn. Animals were then returned to their cages and observed until they achieved sternal recumbency.

### 4.6. DEX Formulation and DEX Periocular Conjunctival Fornix Injection

Each subsequent week for 8 weeks, conscious IOPs were measured and direct ophthalmoscopy was performed. Just as with baseline IOP measurements, all IOP measurements were made between 11:30 am and 1:00 pm, to minimize natural circadian IOP fluctuation issues. Animals were also weighed immediately following IOP measurements. Immediately following weekly IOP measurements and ophthalmoscopic examinations (starting 1 week after IVT injections of Lenti.shRNA.GRβ or Lenti.shRNA.Scramble), animals were given a periocular injection OU of 20 μL of Vehicle solution for DEX (DEX-Veh) (Cohorts 1 (n = 5 animals) and 3 (n = 5 animals)), or micronized DEX suspension at 10 mg/mL concentration (Cohorts 2 (n = 5 animals) and 4 (n = 5 animals)). DEX 10mg/mL suspension or DEX-Veh was formulated as described [20]. Bilateral periocular injections of DEX or DEX-Veh were conducted using our previously described procedure [20]. For each injection, mice were anesthetized with a mixture of isoflurane (2.5%) and O_2(g)_ (0.8 L/min). Both eyes received 1–2 drops of 0.5% proparacaine HCl (Akorn Inc., Lake Forest, IL, USA) for topical anesthesia. The lower lid was then retracted, and the tip of a 32G steel needle attached to a 50 μL volume glass microsyringe (Hamilton Company, Reno, NV, USA) prefilled with DEX suspension or DEX-Veh was inserted through the conjunctival fornix. A 20 μL bolus of DEX suspension was then injected over the course of 10–15 s. Following injection, the needle was rapidly withdrawn. Injections were repeated once per week for a period of 8 weeks.

### 4.7. TM Tissue Isolation

Ocular enucleation and TM tissue isolation was performed at the end of the study under aseptic conditions immediately post-euthanization as described previously [27]. After removal of extraocular tissues from the enucleated eye, the globe was punctured ~1 mm posterior to the limbus using an ocular stab blade. Using the stab-puncture, the globe was hemisected along the posterior limbus, and the resulting anterior segment (containing the TM, ciliary body, iris, lens, and cornea) was further cleaned by removing the iris and ciliary body using a pair of extra-fine forceps and scissors. A 4 mm trephine was then used to cut the cornea out of the corneoscleral segment folded into a semicircle at the center, leaving the TM tissue and the underlying sclera intact. This TM rim tissue was then washed with 1X PBS to remove unwanted cellular or tissue debris. The TM rims from both eyes were then pooled and snap-frozen using liquid nitrogen and stored in a −80 °C freezer for mRNA analysis.

### 4.8. mRNA Isolation and RT-qPCR Analysis

Left and right eye TM rims were pooled together for each mouse, and such preparations from 5 different mice from each group were separately used for total RNA isolation using RNAeasy Micro kit (Qiagen, Germantown, MD, USA) as recommended by the manufacturer protocol. Isolated fibrous TM tissues were mechanically homogenized in the supplied lysis buffer from the kit. For mechanical lysing, two chilled 5 mm stainless steel beads were added to the round-bottom 2 mL microcentrifuge tube with frozen TM rim tissues and lysed in TissueLyser LT (Qiagen, Germantown, MD, USA) at 50 oscillations/s for 15 s for 5 cycles, separated by 1 min ice incubation after each cycle. Eluted RNA was quantified using the NanoDrop 2000 spectrophotometer (ThermoFisher, Waltham, MA, USA). Each RNA preparation was treated with DNase I at room temperature for 15 min, as recommended in the Qiagen RNeasy Micro Kit Protocol (Cat# 74004). SuperScript IV First-Strand synthesis system (ThermoFisher Cat# 18091050) was used to synthesize cDNA using 10 ng of RNA according to the manufacturer protocol. No-RT negative controls were included to detect genomic DNA contamination, as recommended by SuperScript IV First-Strand synthsis sytem (ThermoFisher Cat# 18091050). PCR amplification of the cDNA was performed by qPCR using SYBR Green Supermix (Cat# 172-5271; BIO-RAD). The thermocycling protocol consisted of 10 min at 95 °C, 40 cycles of 15 s at 95 °C, 30 s at 60 °C, and 60 s at 72 °C and finished with a melting curve ranging from 60 to 95 °C to allow distinction of specific products. Mouse GRβ, mouse GRα, and mouse 18S rRNA were amplified in separate reactions. A forward primer in exon 8 (AAAGAGCTAGGAAAAGCCATTGTC) was used in conjunction with a reverse primer in intron 8 (CTGTCTTTGGGCTTTTGAGATAGG) to specifically amplify mGRβ. For mGRα amplification, the same forward primer in exon 8 (AAAGAGCTAGGAAAAGCCATTGTC) was used in conjunction with a different reverse primer in exon 9 for (TCAGCTAACATCTCTGGGAATTCA). 18S rRNA was amplified using the forward primer (TTCGAACGTCTGCCCTATCAA) and the reverse primer (ATGGTAGGCACGGCGACTA). All targets were successfully amplified. Normalization was performed in separate reactions to levels of 18S rRNA.

### 4.9. Statistics

Parametric data are expressed as Mean ± SEM or Mean ± SDM, as stated. Data were tested for normality (Shapiro−Wilk test). IOP data were averaged between left and right eyes within each animal, to avoid confounding effects of lack of independence of two eyes nested within the same animal. Multiple comparisons were conducted by 1- or 2-factor ANOVA (as stated) followed by the Holm–Sidak post hoc test (all pairwise comparisons). TM GRβ mRNA expression (normalized to 18S RNA) in Lenti.shRNA.GRβ- vs. Lenti.shRNA.Scramble-injected groups were compared using the unpaired Student’s *t*-test. Non-parametric (lenticular cataract) data are expressed as median and interquartile range. These data were analyzed using a non-parametric 2-factor ANOVA (Scheirer−Ray–Hare test) followed by Bonferroni’s correction.

Statistical tests were conducted using the software provided by GraphPad Prism 9 or SigmaPlot 15.0 or SAS 9.4. A *p*-value of <0.05 was considered significant.

## 5. Conclusions

We provide further evidence that endogenous GRβ plays a role in modulating the IOP response to DEX in C57BL/6J mice (Figure 6). Knockdown of mRNA-encoding endogenous GRβ results in an OHT response in this species, likely due to reduced total aqueous humor outflow facility. This is significant, since although there are reports on the effects of GC receptor splice variants in other fields, such as inflammatory bowel diseases [59,65,66], cancer biology [67,68,69,70], toxicology [71], gestational diabetes mellitus [72], cell signaling in zebrafish [59,73], effects of cortisol on RACK1 expression [74], and airway diseases such as asthma and chronic sinusitis [66,75], to our knowledge, this is the first study of the individual effects of endogenous GR alternative splice variants on IOP and total aqueous humor outflow facility in GC-OHT.

## Figures and Tables

**Figure 1:**
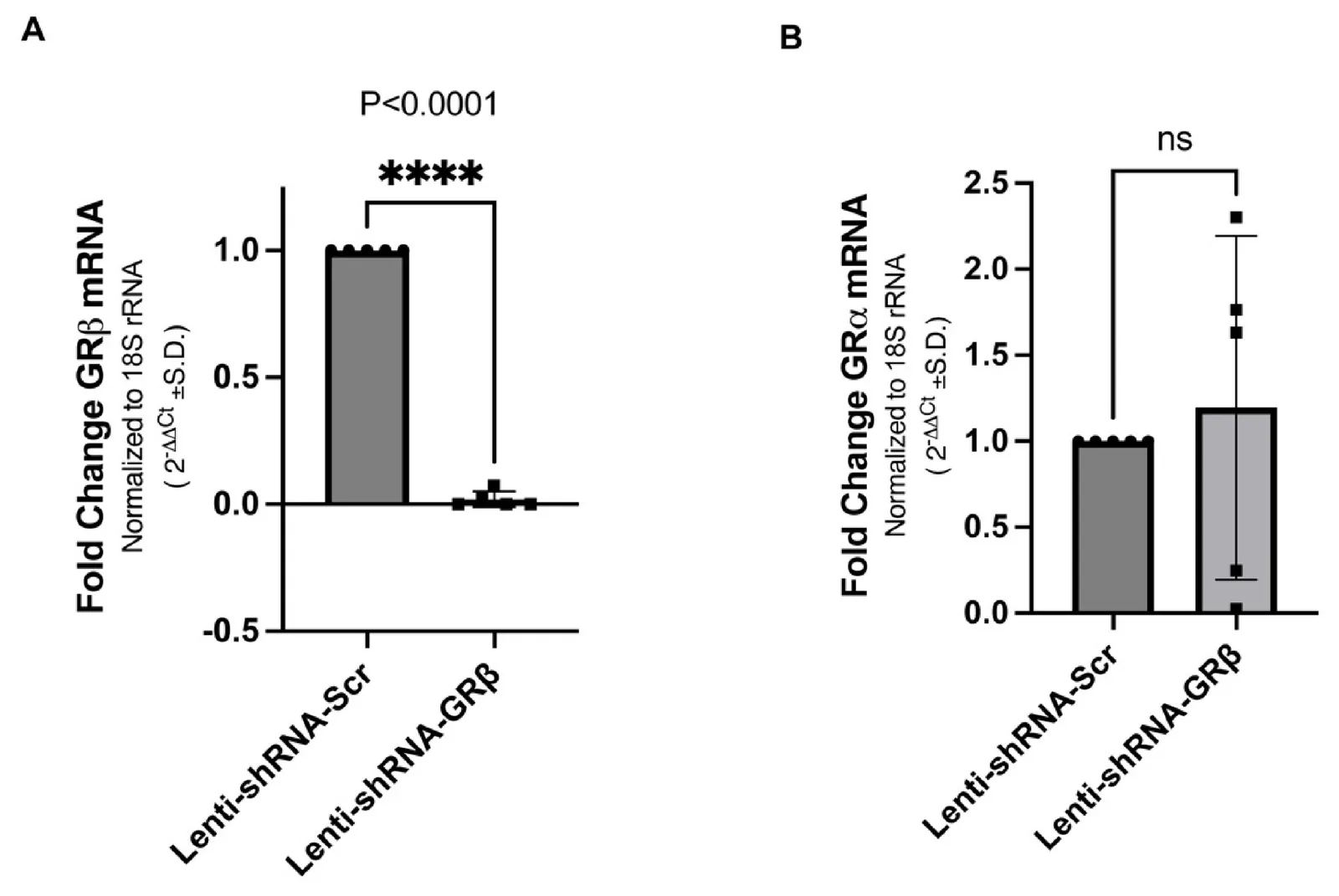
(**A**) Knockdown of GRβ mRNA expression in mouse eyes transduced with Lenti.shRNA.GRβ or control Lenti.shRNA.Scramble vectors. (**A**) QPCR analysis showed that Lenti.shRNA.GRβ treatment caused a significant >95% reduction in GRβ mRNA expression (normalized to 18S RNA expression) in TM tissues (OD and OS eyes of each mouse combined; n = 5 mice/group). **** *p* < 0.0001, unpaired *t*-test (2 tailed). (**B**) GRα mRNA levels normalized to 18S RNA expression. Lenti.shRNA.GRβ treatment did not cause a significant change (ns) in GRα mRNA levels. (n = 5 mice/group). *p* = 0.647, unpaired *t*-test (2 tailed).

**Figure 2:**
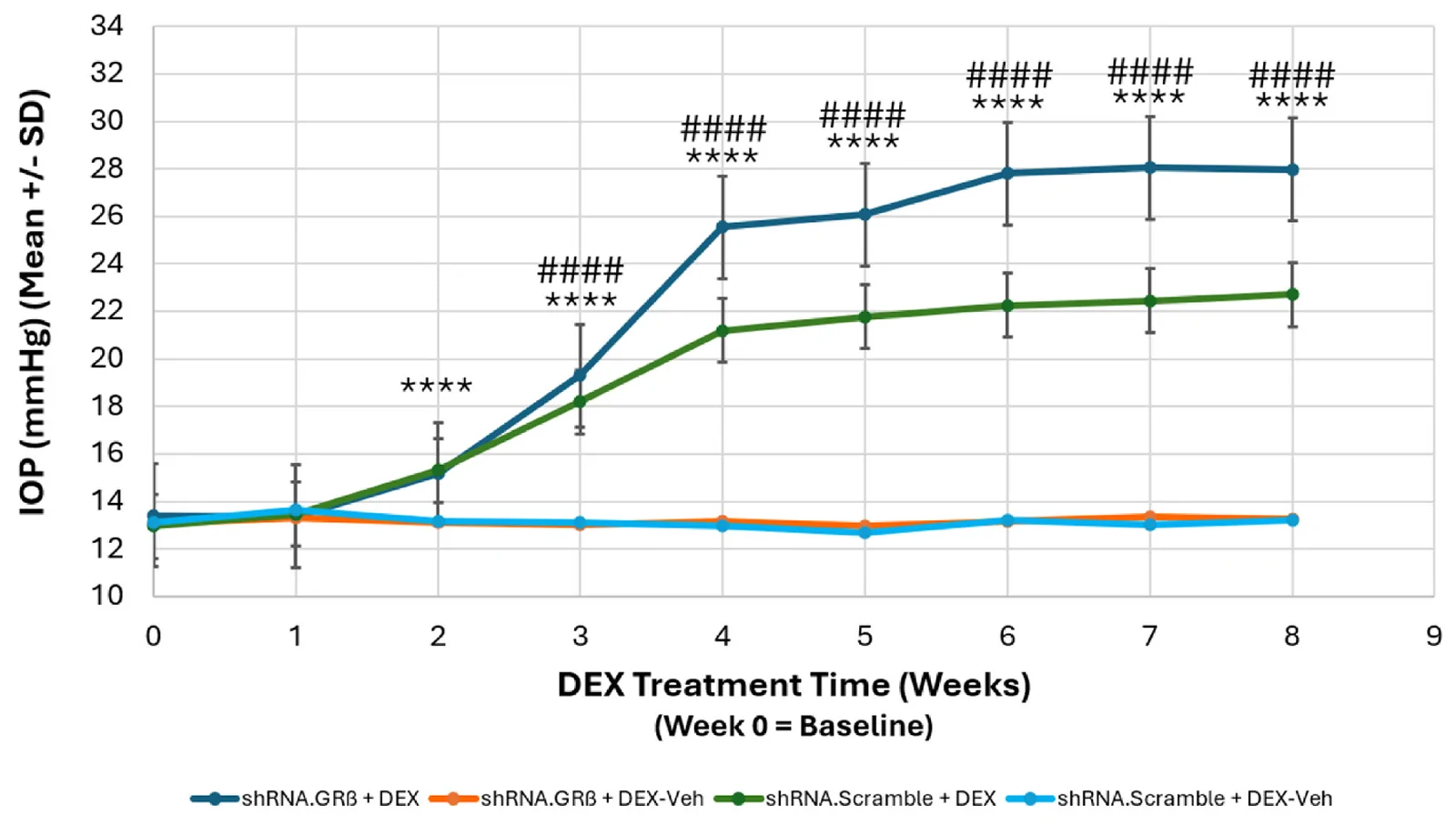
Conscious intraocular pressure (IOP) (mean +/− SD) in C57BL/6J mice injected OU with Lenti.shRNA.GRβ or Lenti.shRNA.Scramble (Week 0) followed by weekly (Weeks 1–8) DEX or Veh periocular Injections. **** *p* < 0.0001; significance of difference of DEX groups from DEX-Veh groups as indicated by 2-factor ANOVA followed by Holm−Sidak post hoc test. #### *p* < 0.0001; significance of difference of DEX + shRNA.GRβ group from DEX + shRNA.Scramble group as indicated by 2-factor ANOVA followed by Holm–Sidak post hoc test.

**Figure 3:**
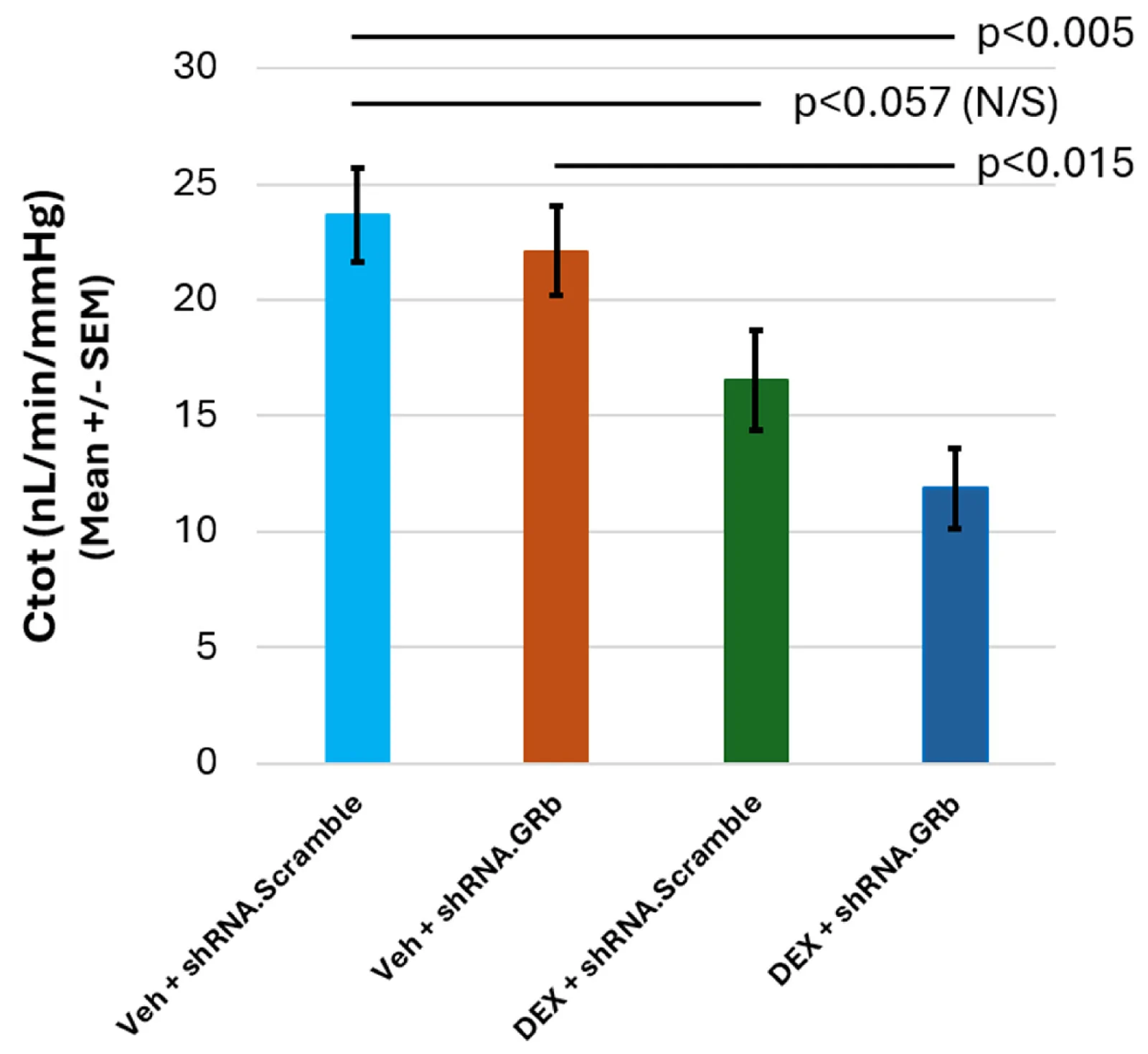
Total aqueous humor outflow facilities (C_tot_) of C57BL/6J mice injected OU with Lenti.shRNA.GRβ or Lenti.shRNA.Scramble (Week 0) followed by weekly (Weeks 1–8) DEX or DEX-Veh periocular Injections. Outflow was measured in live anesthetized mice after 8 weeks of treatment. Means of treatment groups significantly different as indicated by 1-factor ANOVA (*p* = 0.002). Individual *p*-values indicated generated by Holm–Sidak pairwise post hoc comparisons. The apparent differences between the two shRNA.Scramble groups just missed statistical significance (N/S). The apparent DEX-induced further decrease C_tot_ in the GRβ knockdown group also was not statistically significant.

**Figure 4:**
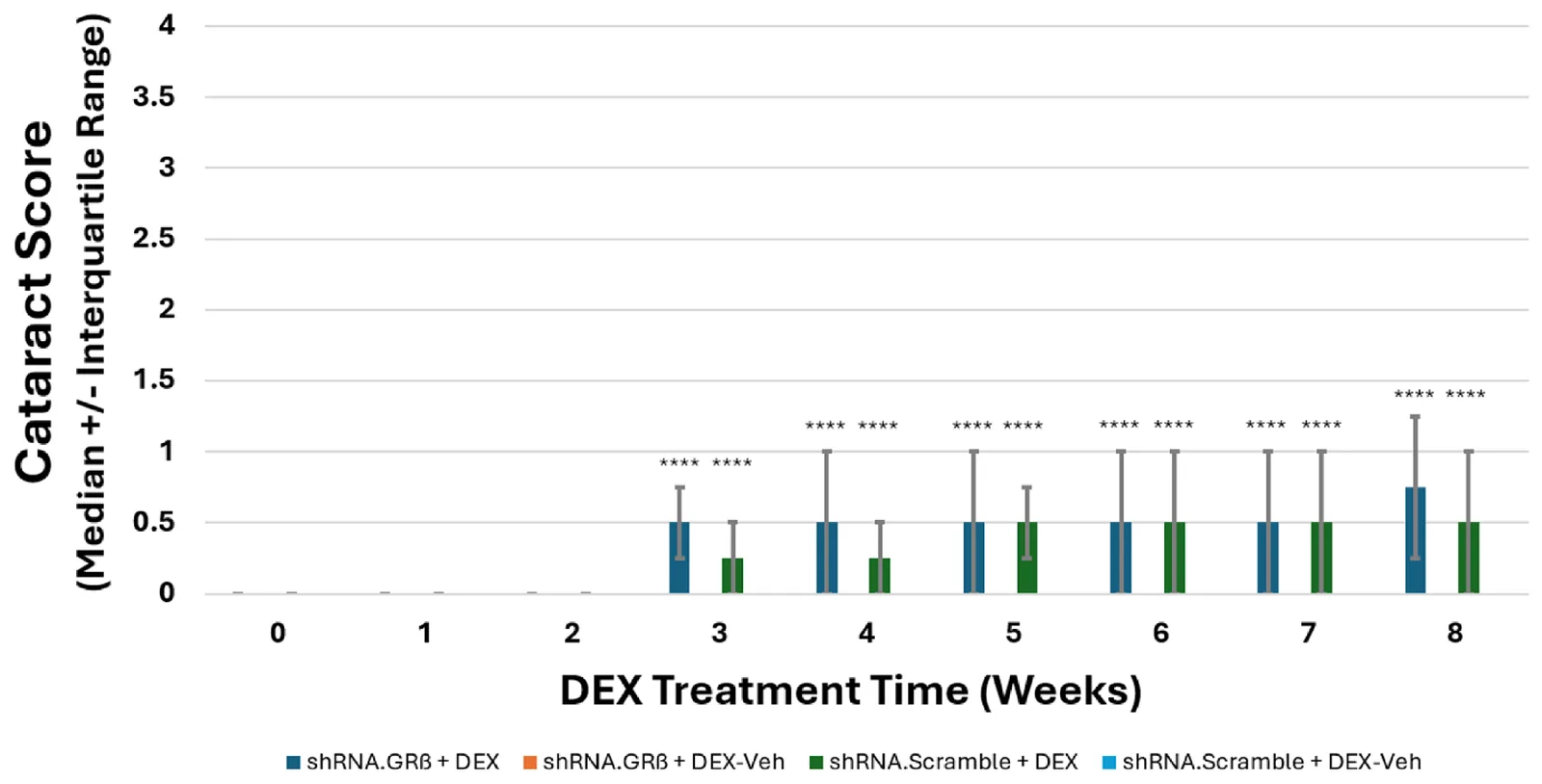
Development of mild lenticular cataracts following 8 weeks of periocular DEX treatment. Cataracts were scored using direct ophthalmoscopy according to the criteria specified in Table 1. The development of mild DEX-induced cataracts was statistically significant from 3–8 weeks compared to vehicle-treated animals (n = 5/group; **** *p* < 0.0001) as indicated by Scheirer−Ray–Hare non-parametric 2-factor ANOVA by ranks followed by Bonferroni’s correction.

**Figure 5:**
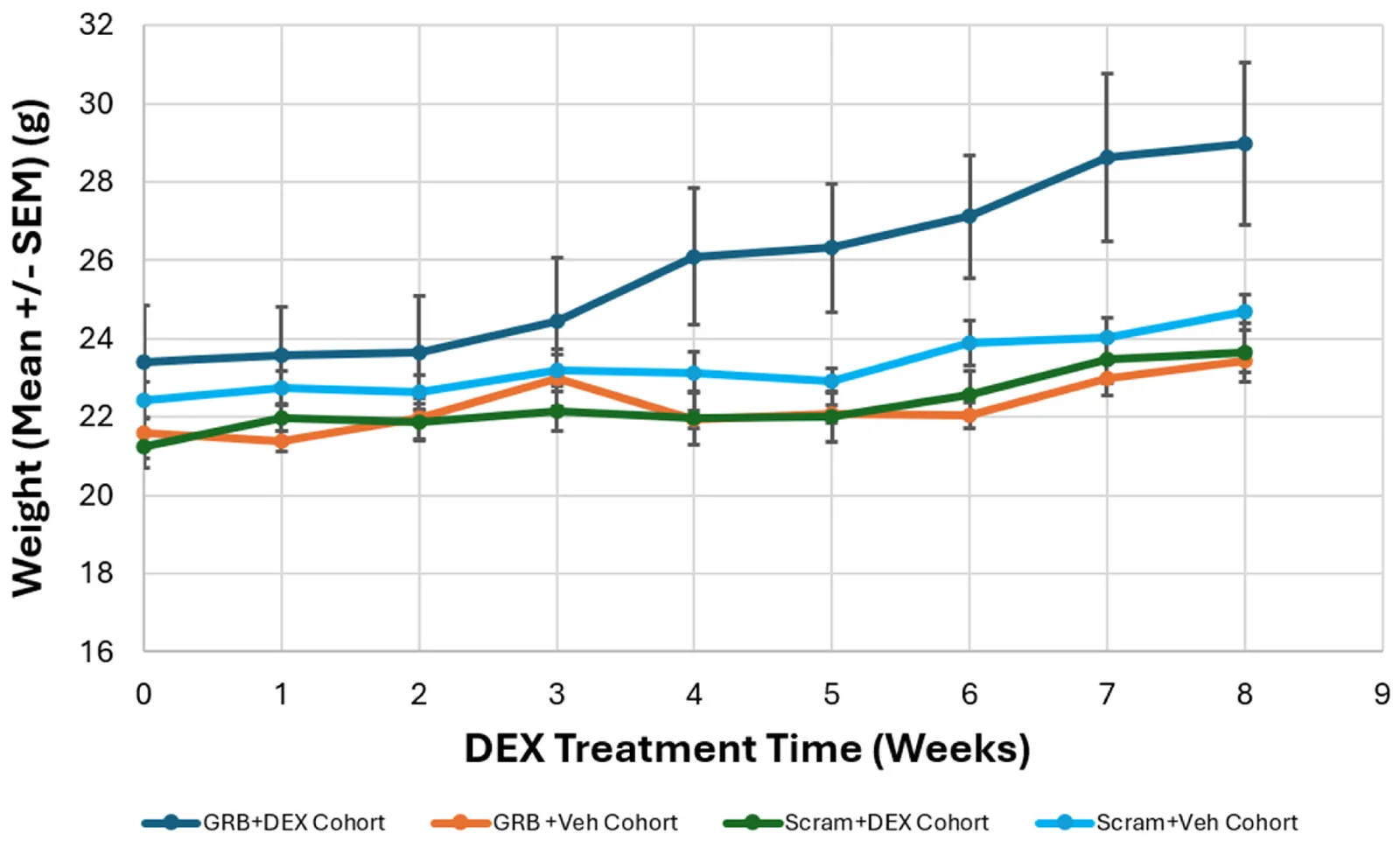
Body weights in C57BL/6J mice injected OU with Lenti.shRNA.GRβ or Lenti.shRNA.Scramble (Week 0) and then weekly (Weeks 1–8) DEX or DEX-Veh periocular injections. No animal lost ≥ 10% of body weight during the entire study period.

**Figure 6:**
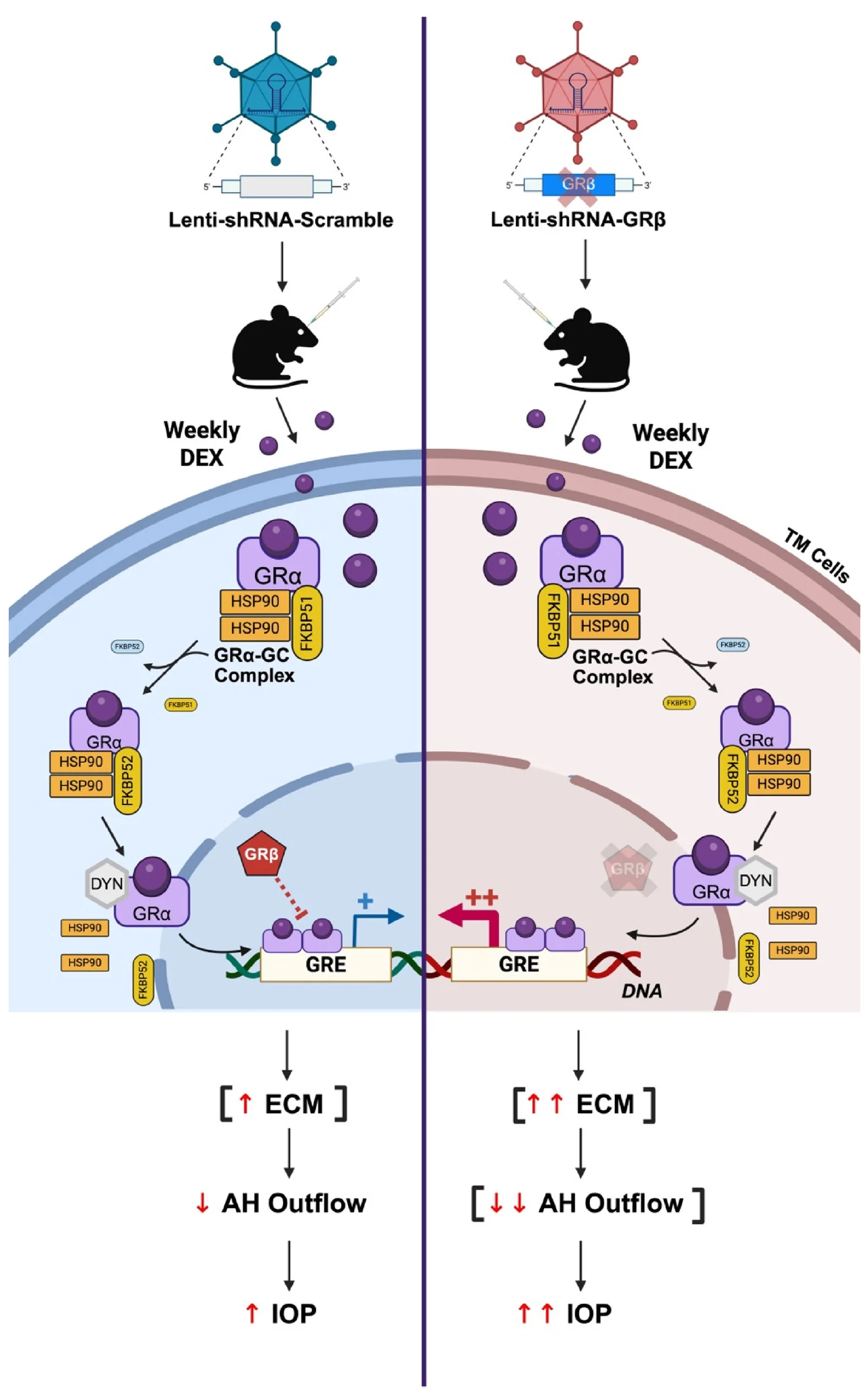
**Summary:** Effect of knocking down endogenous GRβ in the mouse TM on the AH outflow facility and IOP. Mouse eyes were transduced with lentiviral vectors encoding shRNA. Scramble (non-targeting control) (**left side**) or shRNA.GRβ (to knockdown GRβ in the TM) (**right side**). The mice then received weekly periocular injections of DEX, and IOPs were recorded weekly in conscious mice for a total of 8 weeks. AH outflow facilities were recorded in live anesthetized mice at the end of the study. Although not directly shown in this study, DEX increased the deposition of ECM material within the TM [34,35,36,49] in other models. Therefore, we have added “[ ]” to indicate our mechanistic hypothesis that ECM also is elevated in our study. This among other effects decreased the AH outflow facility and elevated IOP. Although we have shown further decreased AH outflow in the GRβ knockdown group, this was not statistically significant, hence the added “[ ]”. Endogenous GRβ within the TM partially tempered these DEX effects (**left side**), while knockdown of endogenous GRβ (**right side**) exacerbated these DEX effects causing additional decreased AH outflow and enhanced IOP elevation. (Figure made using BioRender).

**Figure 7:**
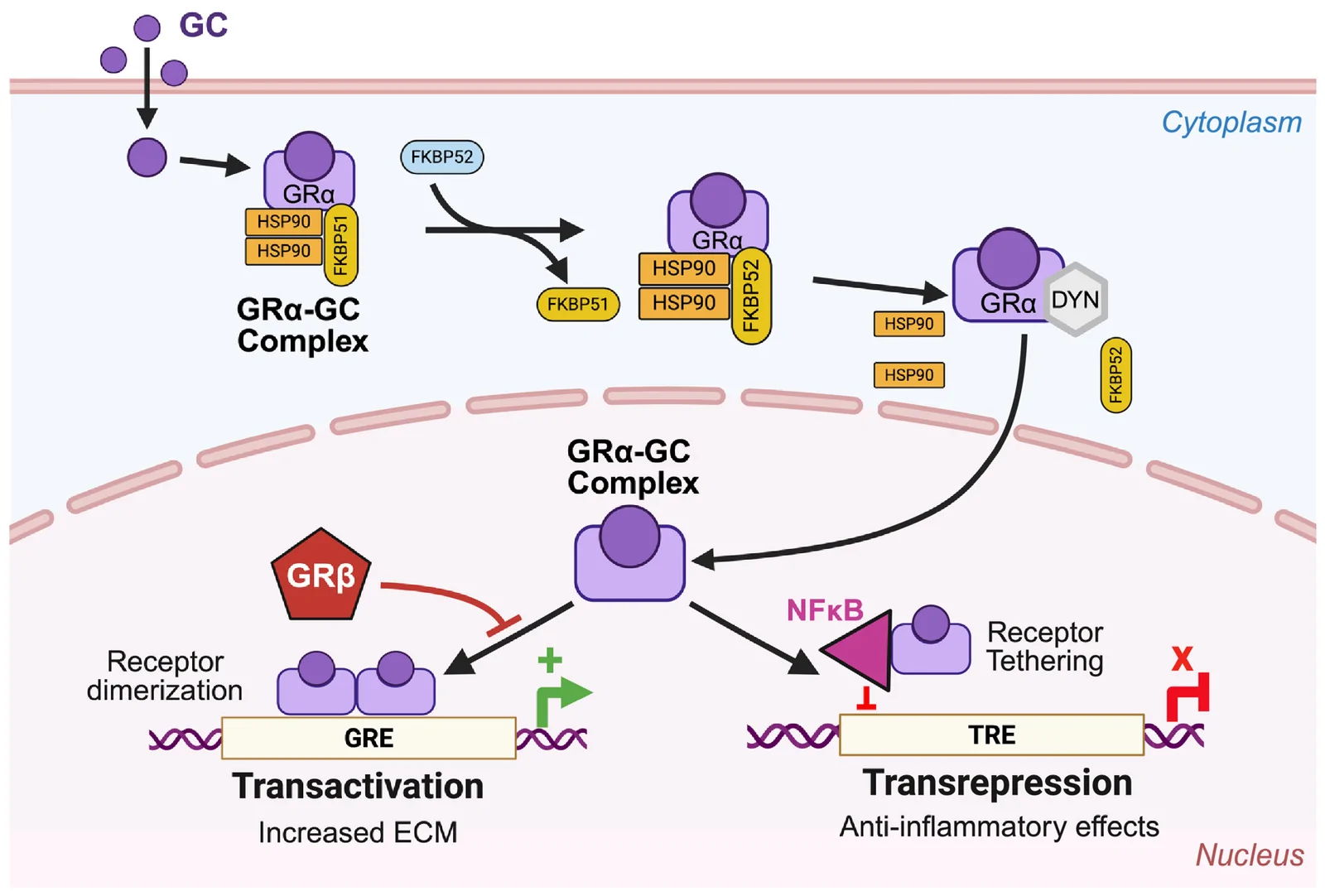
Summary of GC mechanism of action. GCs bind to a GRα complex containing GRα/HSP90/FKBP1. GRα undergoes a conformational change initially exchanging accessory proteins with the microtubule associated protein DYN2, and the activated GRα-GC is transported to the nuclear envelope, where it enters the nucleus via nuclear pores. Within the nucleus, GR alters gene expression in three different ways. (1) During transactivation, GRα-GC homodimerizes and binds to glucocorticoid response elements (GREs) within responsive genes, where it directly regulates gene expression. GC-induced ECM accumulation within the TM as well as GC-OHT are mediated via transactivation. (2) In transrepression, activated GRα-GC monomers bind to other transcription factors (e.g., NFκB) preventing these transcription factors from binding response elements (TREs) within the genes they regulate, thereby blocking gene expression. Many of the anti-inflammatory and immunosuppressive effects of GCs are mediated by GR transrepression. (3) The alternatively spliced isoform GRβ does not bind GCs, resides within the nucleus, and acts as a dominant negative regulator of GRα activity. GRβ suppresses GRα mediated transactivation, although it is unclear (and may be context dependent) whether GRβ also blocks GRα transrepression. (Figure made using BioRender).

**Table 1:** The standardized ordinal grading scale used to assess lenticular cataract severity in live mice during direct ophthalmoscopic observations. Lenticular cataract assessments were observer subjective. We developed this scale for our own use; to our knowledge it is not published elsewhere. All cataracts observed were nuclear or cortical. No posterior subcapsular cataracts were observed.

Lenticular Cataract Score	Appearance of Crystalline Lens by Direct Ophthalmoscopy
0	Normal appearance. No opacities observed
0.5	Barely visible discrete single nuclear or cortical cataract
1	More readily visible small discrete nuclear or cortical opacity, or 2–3 barely visible nuclear and/or cortical opacities.
2	Larger nuclear or cortical opacity, or 2–3 small nuclear and/or cortical opacities, involving ~20% to ~50% of the lens.
3	Mature cataract, involving entire lens nucleus and cortex.
4	Severe mature cataract, involving entire lens nucleus and cortex. Lens presents dried, hard, and crusted appearance.

## Data Availability

The original contributions presented in this study are included in the article/supplementary material. Further inquiries can be directed to the corresponding author.

## Supplementary Materials

The following supporting information can be downloaded at: https://www.mdpi.com/article/10.3390/ijms27198483/s1.
