# Supplementary material for: Knock-Down of Glucocorticoid Receptor Beta (GRβ) Enhances the Ocular Hypertensive Response to Dexamethasone in C57BL/6J Mice
Source: Int J Mol Sci. 2026 Sep 23;27(19):8483. doi: 10.3390/ijms27198483 (PMC13664864; doi:10.3390/ijms27198483)
Supplement: Supplementary file 1 [file ijms-27-08483-s001.zip › ijms-4416597-supplementary.pdf]

*Supplementary Material*

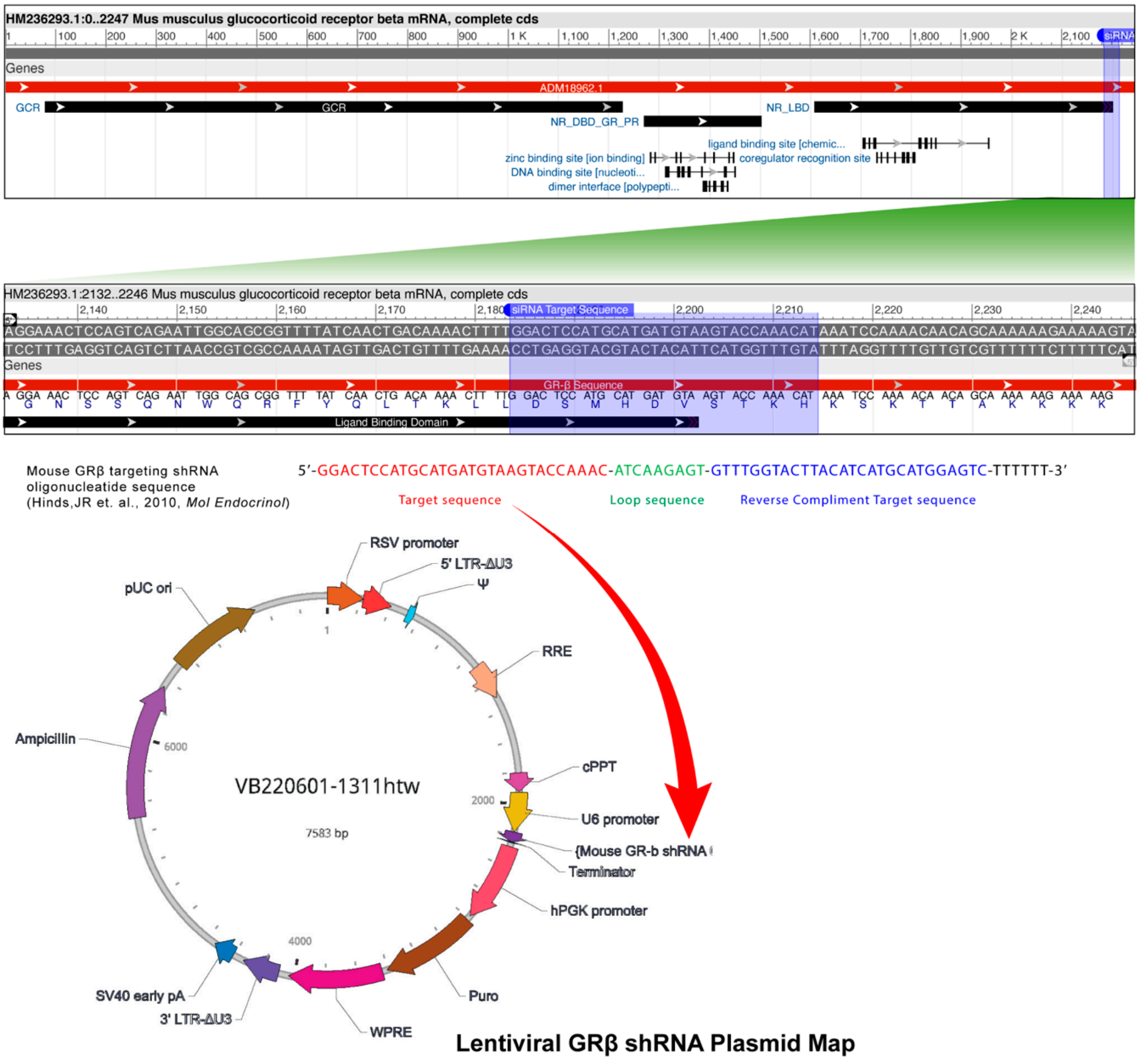


**Supplementary Figure S1.** Alignment of the lentiviral shRNA sequence targeting GRβ mRNA sequence beginning in exon 8 (ligand binding domain) and extending into intron 8. Bottom: The corresponding plasmid map of the Lenti.shRNA. GRβ vector.

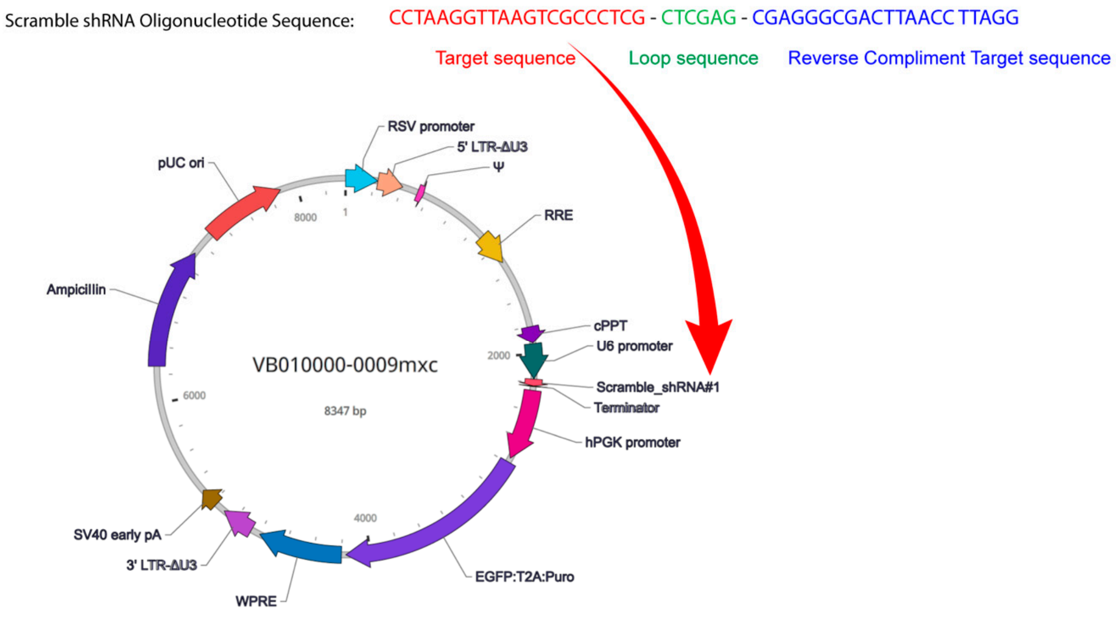


Control Lentiviral shRNA Plasmid Map

**Supplementary Figure S2.** Plasmid map for the control vector Lenti-shRNA-Scramble with the scramble shRNA sequence.
